# Distinct loiasis infection states and associated clinical and hematological manifestations in patients from Gabon

**DOI:** 10.1371/journal.pntd.0010793

**Published:** 2022-09-19

**Authors:** Luzia Veletzky, Kirsten Alexandra Eberhardt, Jennifer Hergeth, Daniel Robert Stelzl, Rella Zoleko Manego, Ghyslain Mombo-Ngoma, Ruth Kreuzmair, Gerrit Burger, Ayôla Akim Adegnika, Selidji Todagbe Agnandji, Pierre Blaise Matsiegui, Michel Boussinesq, Benjamin Mordmüller, Michael Ramharter

**Affiliations:** 1 Department of Medicine I, Division of Infectious Diseases and Tropical Medicine, Medical University of Vienna, Vienna, Austria; 2 Department of Tropical Medicine, Bernhard Nocht Institute for Tropical Medicine & I. Dep. of Medicine, University Medical Center Hamburg-Eppendorf, Hamburg, Germany; 3 Centre de Recherches Médicales de Lambaréné, Lambaréné, Gabon; 4 German Center For Infection Research (DZIF), Hamburg-Borstel-Riems, Germany; 5 Department of Urology, University Medical Center Hamburg-Eppendorf, Hamburg, Germany; 6 Institute of Tropical Medicine, University of Tübingen, Tübingen, Germany & German Center for Infection Research, partner site Tübingen, Tübingen, Germany; 7 Centre de Recherches Médicales de la Ngounié, Fougamou, Gabon; 8 Institut de Recherche pour le Développement (IRD), UMI 233-TransVIHMI-Inserm U1175-University of Montpellier, Montpellier, France; 9 Radboud University Medical Center, Department of Medical Microbiology, Nijmegen, The Netherlands; University of Zurich, SWITZERLAND

## Abstract

**Background:**

Loiasis–a filarial disease endemic in Central and West Africa–is increasingly recognized as significant individual and public health concern. While the understanding of the disease characteristics remains limited, significant morbidity and excess mortality have been demonstrated. Here, we characterize clinical and hematological findings in a large cohort from Gabon.

**Methods:**

Loiasis-related clinical manifestations and microfilaremia, hemoglobin and differential blood counts were recorded prospectively during a cross-sectional survey. For analysis, participants were categorized into distinct infection states by the diagnostic criteria of eye worm history and microfilaremia.

**Results:**

Analysis of data from 1,232 individuals showed that occurrence of clinical and hematological findings differed significantly between the infection states. Eye worm positivity was associated with a wide range of clinical manifestations while microfilaremia by itself was not. *Loa loa* infection was associated with presence of eosinophilia and absolute eosinophil counts were associated with extent of microfilaremia (p-adj. = 0.012, ß-estimate:0.17[0.04–0.31]).

**Conclusions:**

Loiasis is a complex disease, causing different disease manifestations in patients from endemic regions. The consequences for the affected individuals or populations as well as the pathophysiological consequences of correlating eosinophilia are largely unknown. High-quality research on loiasis should be fostered to improve patient care and understanding of the disease.

## Author summary

Loiasis is a parasitic disease, endemic in parts of Western and Central Africa. While the disease has traditionally been considered to be benign, only recently significant disease morbidity and mortality have been shown. Most of the knowledge about loiasis, however, stems from reports on returning travelers, while comprehensive data from patients living in endemic areas are missing. Blood microfilaremia and reported eye worm are important diagnostic manifestations of the disease, but they can occur independent of each other in affected individuals. We analyzed hematological and clinical findings according to loiasis infection states, comprising reported eye worm and microfilaremia positivity, in a large group of individuals from a highly endemic area.

While we found that all loiasis infection states were significantly associated with absolute blood eosinophilia, the eosinophilia was more pronounced in microfilaremic loiasis. Further, there was an association between the extent of microfilaremia and absolute eosinophilia. Analyzing the frequency of clinical disease manifestations, we found that eye worm positive loiasis was associated with a range of symptoms, but microfilaremic loiasis was not. Summarizing, even in highly endemic populations different loiasis infection states are associated with distinct disease manifestations, underlining that loiasis is a versatile and indeed relevant disease.

## Introduction

Loiasis, caused by the filaria *Loa loa*, is a parasitic disease endemic in rural West and Central Africa [1]. It is transmitted by tabanid flies of the genus *Chrysops* and 14 million people are estimated to be infected. In hyperendemic regions prevalence often exceeds 60% in adults [2]. Loiasis is commonly known for the pathognomonic sign of the “eye worm”, which is caused by adult *L*. *loa* filariae transiently migrating under the patient’s conjunctiva. This distinct clinical feature has been used as an epidemiological marker to assess community prevalence of loiasis in endemic settings (“RAPLOA” survey) [2].

Adult *L*. *loa* filariae can live more than 15 years in their definitive hosts, continuously migrating through the soft tissue and reproducing sexually. Female worms release microfilariae (mf) which can be found in the peripheral blood of patients, in sometimes very high numbers (up to 300,000 mf/mL) [1,3]. Loiasis without detectable microfilaremia is commonly referred to as “occult loiasis”. Determinants for the presence or level of microfilaremia are incompletely understood and may include host genetics, varying immune responses, parasite factors such as number of adult worms, worm fecundity or sex ratio [4–6]. The majority of clinical data in scientific literature stem from reports on travelers and temporary residents of endemic regions [1,7–11]. Only few studies systematically evaluated the clinical picture of loiasis in long-term residents of endemic regions, not least due to the supposedly benign disease course [4,12–17]. Chronic infection has been associated with unspecific manifestations, such as itching, body pains or fatigue and importantly, individuals with high microfilarial densities are known to be at risk for spontaneous or treatment-related severe manifestations and excess mortality [1,7,12,13,18,19]. Eosinophils are known to be important immune-effector cells in parasitic infections and an association between loiasis and eosinophilia has been described in short term residents, but their role in chronic loiasis has barely been studied [8,9,12].

While loiasis is in general increasingly understood as an infection of clinical relevance, many questions on the clinical features and manifestations in the most affected populations are still unanswered. To improve our understanding of the disease, we evaluated clinical and hematological findings associated with loiasis in a large cohort of individuals living in rural Gabon [18].

## Methods

### Ethics statement

The study was conducted in accordance with the Declaration of Helsinki, ICH-GCP guidelines and local regulations. Ethical approval was obtained from the Comité d’Ethique Institutionnel du Centre de Recherches Médicales de Lambaréné; IORG0007336/IRB00008812. Project aims and procedures were presented to local authorities, who agreed to all study-related activities. All individuals above one year of age were invited to participate and study-related procedures were only initiated after provision of written informed consent by participants or their legal representatives.

### Data collection

Detailed methods of data collection were described previously [18]. In brief, data were gathered in a cross-sectional survey conducted in 2017–2018 in a rural region of Gabon where *L*. *loa* is endemic [3,20]. A standardized questionnaire was used to collect baseline demographic data and loiasis-related complaints, including the RAPLOA questionnaire to inform on life-time and within the last year eye worm history [2]. 5 mL of peripheral blood were collected from all participants between 10 am and 3 pm in an EDTA tube. Samples were handled on the same day following standardized procedures.

### Diagnostics

Microfilaria diagnostics included thick blood smears and concentration technique which have been described in detail before [18]. Two Giemsa-stained thick blood smears of 10 μL each were examined under a microscope and, if no mf were found, additional examination of 1 mL whole blood was performed after lysis and centrifugation. *L*. *loa* and *M*. *perstans* mf were distinguished by trained microscopists based on size, head and tail characteristics [21]. Hemoglobin levels and white blood cell (WBC) counts were measured using rapid diagnostic devices (HemoCue WBC System, HemoCue Hb 201+) or Pentra hematology analyzers. Results of both machines were compared in a subgroup and shown to be comparable.

*M*. *perstans* and *M*. *sp*. *Deux* are co-endemic in the study area. As microfilarial densities are often low in mansonellosis, DNA was extracted from 500 μl whole blood and analyzed by polymerase chain reaction (PCR) to increase diagnostic sensitivity [3]. Detailed methods are provided in the supporting information.

### Statistical analysis

Statistical analysis was performed using the software package R (Version 4.0.5, R Foundation for Statistical Computing, Vienna, Austria). Participants were categorized into “infection states”, defined by presence/absence of eye worm history and presence/absence of detectable microfilaremia. Thus, the main *L*. *loa* infection states were: 1) No sign of *L*. *loa* infection, i.e. absence of microfilaremia and no history of eye worm (LN), 2) detectable microfilaremia but no history of eye worm (MF), 3) positive history of eye worm but no detectable microfilaria (EW) and 4) history of eye worm and detectable microfilaremia (EWMF). To assess differences according to the level of *L*. *loa* microfilaremia, microfilaremic participants were categorized into three groups: low (1–7,999 mf/mL; LMF), high (8,000–19,999 mf/mL; HMF) and hyper-microfilaremia (≥20,000 mf/mL; HYMF) (see Table 1). Occurrence of reported symptoms during the previous three months and hematological parameters were analyzed and compared between infection states. Calabar swelling was not considered as diagnostic criterion as it has been shown to be of limited specificity but was included as symptom in data analysis if it occurred during the previous year [2].

**Table 1:** Definitions of the classified infection states described in the paper.

Classified infection state	Diagnostic criterion	Abbreviation
Eye worm positive loiasis	Eye worm positivity based on the standardized RAPLOA questionnaire [2,42]	EW
Microfilaremic loiasis	Detectable midday microfilaremia assessed by microscopy of two Giemsa-stained thick blood smears (total of 20μl full blood) AND 1mL of full blood assessed after hemolysis using saponin followed by centrifugation	MF
Eye worm positive as well as microfilaremic loiasis	Individuals fulfilling both criteria	EWMF
Microfilaremic loiasis with low microfilaremia	Microfilaremia between 1–7,999 mf/mL	LMF
Microfilaremic loiasis with high microfilaremia	Microfilaremia between 8,000–19,999 mf/mL	HMF
Microfilaremic loiasis with hyper microfilaremia	Microfilaremia above ≥20,000 mf/mL	HYMF
No sign of loiasis infection	Individuals who have no history of eye worm and no detectable microfilaremia	LN

Categorical variables were compared using the χ^2^ test or the Fisher exact test as appropriate. Multi-group comparisons were performed and corrected for multiple comparisons using the false discovery rate method. Continuous variables were expressed as median and interquartile range (IQR). To correct for demographic parameters and filarial co-infections, multiple regression models were run and adjusted for the following possible confounders: age group, sex and presence of *M*. *perstans* microfilariae. In case of logistic regression analysis, adjusted prevalence ratios (aPR, 95% confidence interval, CI) were computed.

## Results

### Study population

Data on history of eye worm migration and microfilaremia were available for 1,232 participants. First, the cohort was categorized into the above described four mutually exclusive infection states LN, MF, EW and EWMF. MF, EW and EWMF are thus the subgroups of individuals with definite signs for loiasis (LP).

Within the loiasis positive infection states (LP, n = 626), EW was the most common infection state (52.4%, n = 328), followed by EWMF (30.7%, n = 192) and MF (16.9%, n = 106) (see Table 2). Inter-group comparisons between the infection states revealed significant differences between age and sex distribution. There was no difference in the distribution of extent of microfilaremia between women and men, nor between the adult age groups when comparing the microfilaremic categories (see Table 3).

**Table 2:** Distribution of subjects according to the infection state within each age and sex category and comparisons of these distributions between infection state categories. Distributions by age categories were compared using the Fisher’s exact test, and distributions by sex were compared using the χ2 test.

Age and sex distributions of loiasis infection states																	
	Whole cohort		LN*		MF*		EW*		EWMF*		p-value	Post hoc test (False discovery rate correction)					
	N	%°	N	%	n	%	N	%	n	%	overall	Inter-group comparisons					
**Total**	1232	100	606	49.2	106	8.6	328	26.6	192	15.6		**MF vs. LN**	**EW vs. LN**	**EWMFvs. LN**	**EW vs. MF**	**EWMFvs. MF**	**EW vs. EWMF**
**Age** (years)																	
<15	157	12.7	134	85.3	6	3.8	15	9.6	2	1.3							
15–59	751	61.0	364	48.5	57	7.6	212	28.2	118	15.7	<0.001	<0.001	<0.001	<0.001	0.125	0.055	0.048
≥60	324	26.3	108	33.3	43	13.3	101	31.2	72	22.2							
**Sex**																	
Female	668	54.2	343	51.4	31	4.6	209	31.3	85	12.7	<0.001	<0.001	0.041	0.006	<0.001	0.019	<0.001
Male	564	45.8	263	46.6	75	13.3	119	21.1	107	19.0							

* LN = No sign of loiasis infection; MF = detectable microfilaremia but no history of eye worm; EW = positive history of eye worm but no detectable microfilaria; EWMF = positive history of eye worm as well as detectable microfilaremia, %° = column percentages, % = row percentages

**Table 3:** Distribution of subjects according to their microfilaremia category within each age and sex category and comparisons of these distributions between microfilaremia categories. Distributions by age and sex categories were compared using the Fisher’s exact test.

Age and sex distributions of subgroups of microfilaremic individuals											
	N	LMF*		HMF*		HYMF*		p-value	Post hoc test (False discovery rate correction)		
		n	%	n	%	n	%	overall	Inter-group comparisons		
**Total**	298	242	81.2	43	14.4	13	4.4		**LMF vs. HMF**	**LMF vs. HYMF**	**HMF vs. HYMF**
**Age** (years)											
<15	8	8	100	0	0	0	0				
15–59	175	140	80.0	28	16.0	7	4.0	0.768	0.795	0.852	0.795
≥60	115	94	81.7	15	13.0	6	5.2				
**Sex**											
Female	116	96	82.8	16	13.8	4	3.5	0.808	0.866	0.866	0.866
Male	182	146	80.2	27	14.8	9	5.0				

### Loiasis infection states and clinical presentation

**Fig 1:**
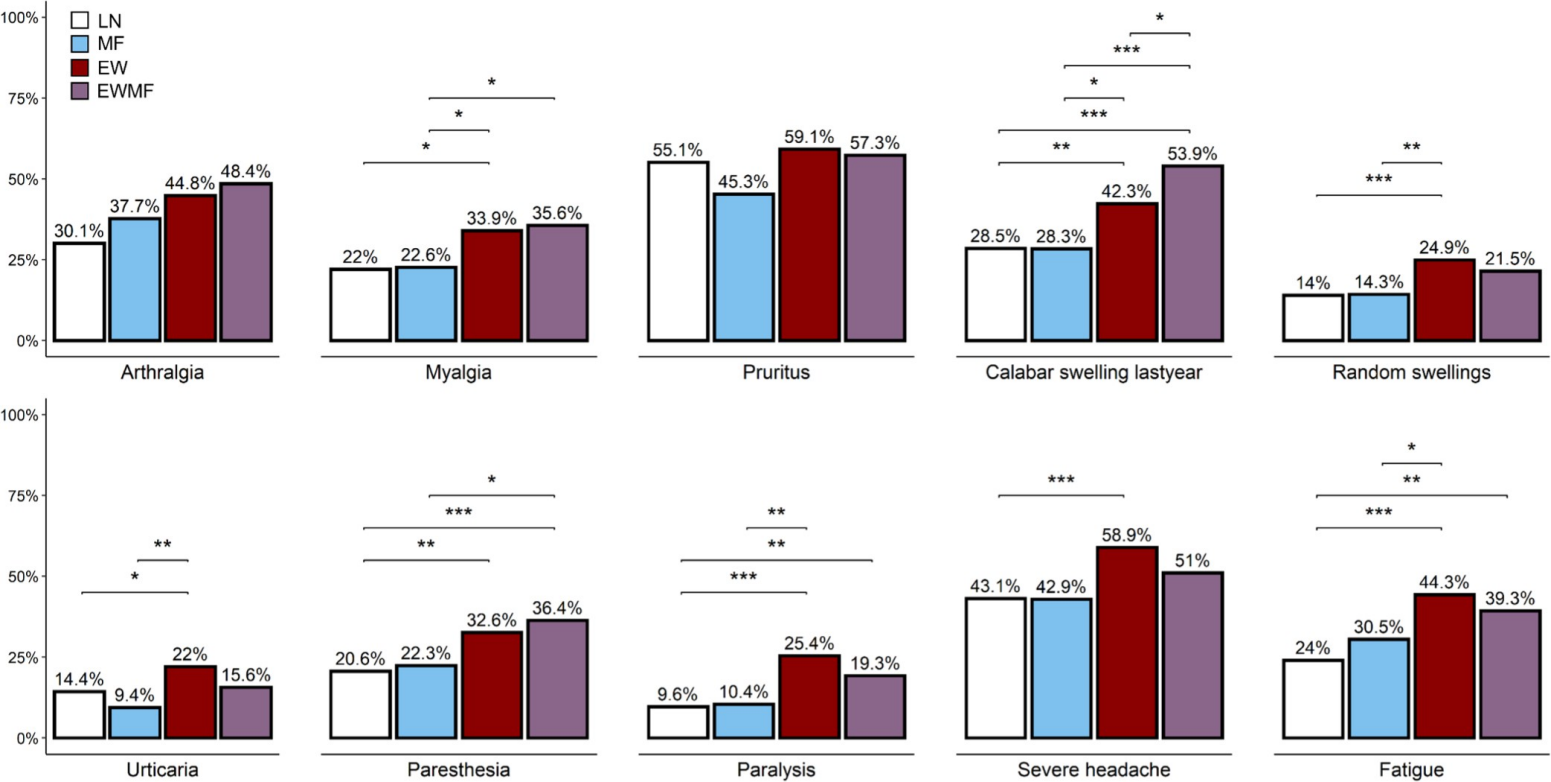
Frequencies of signs and symptoms within the three loiasis infection states and in uninfected individuals as bar plots and adjusted p-values of inter group comparisons, if significant. All intergroup comparisons are adjusted to sex, age and *Mansonella* PCR. * p-adj. <0.05; **p-adj. <0.01; *** p-adj. <0.001; * LN = No sign of loiasis infection; MF = detectable microfilaremia but no history of eye worm; EW = positive history of eye worm but no detectable microfilaria; EWMF = positive history of eye worm as well as detectable microfilaremia.

Occurrence of the disease manifestations were compared between the infection states and results were adjusted to possible confounding factors including sex, age and *M*. *perstans* microfilaremia (see Tables A, B, C in S1 Appendix). Fig 1 displays the frequency of reported manifestations by infection state, and adjusted p-values of intergroup comparisons if significant. In comparison to LN, each of the two groups with history of eye worm (EW and EWMF) reported several signs and symptoms significantly more often. This was the case for Calabar swelling-like manifestations (adjusted prevalence ratio [95% confidence interval]: (1.40 [1.10–1.79] for EW and 2.05 [1.56–2.69] for EWMF), paresthesia (1.51 [1.19–1.92] and 1.73 [1.32–2.26]), transient paralysis of extremities (2.63 [1.86–3.72] and 1.78 [1.15–2.76]), and fatigue (1.81 [1.43–2.30] and 1.49 [1.12–2.01]). In addition, individuals in the EW category reported myalgia and severe headache more often compared to LN (1.36 [1.01–1.82] and 1.33 [1.15–1.54], respectively). The frequency of the reported manifestations was similar between MF and LN subjects.

Comparing the 3 LP categories among each other, EW and EWMF subjects reported some manifestations more frequently than MF individuals. This was the case for myalgia (1.79 [1.08–2.97] and 1.76 [1.04–2.99], respectively) and Calabar swelling-like manifestations (1.55 [1.00–2.40] and 2.27 [1.45–3.55]). Further, EW subjects reported several disease manifestations more often than the MF subjects; this was the case for transient swellings of any body part (other swellings 1.81 [1.05–3.10]), transient paralysis of extremities (2.84 [1.48–5.47]), urticaria (2.38 [1.23–4.59]) and fatigue (1.53 [1.02–2.30]). Comparing the EW and EWMF categories, patients in the EWMF group reported significantly fewer Calabar swelling-like manifestations (0.68 [0.51–0.91]), whereas patients in the EW category indicated more often urticaria (p = 0.062, 1.48 [0.97–2.24]). HMF and HYMF individuals reported more often an episode of eye worm migration during the previous year compared to LMF (58.2% versus 35.5%; p-adj. = 0.003).

In summary, the infection states comprising participants with history of eye worm were significantly associated with the occurrence of clinical complaints. Contrarily, microfilaremic individuals reported complaints in a similar proportion as persons without signs of loiasis infection. The proportion of reported manifestations was independent of extent of microfilaremia, with exception for occurrence of eye worm.

### Loiasis infection states and hematological phenotypes

**Fig 2:**
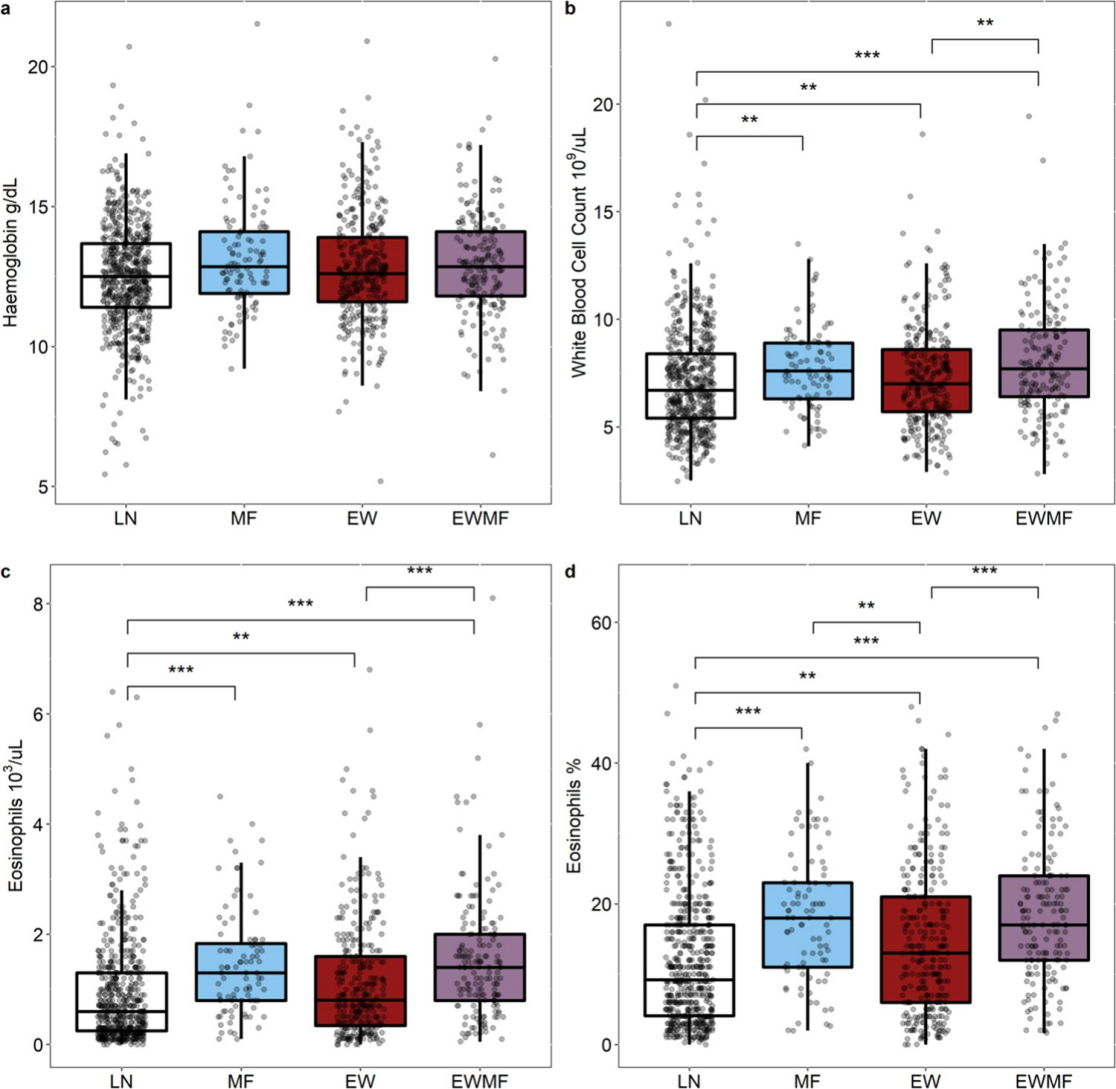
**Distribution of hematological findings including median and interquartile range of hemoglobin (A), total white blood cell count (B), absolute (C) and relative eosinophil counts (D) within the four infection states.** Adjusted p-values of intergroup comparisons are provided if <0.05. All intergroup comparisons are adjusted to sex, age and positivity of *Mansonella* PCR. * p-adj. <0.05; **p-adj. <0.01; *** p-adj. <0.001; LN = No sign of loiasis infection; MF = detectable microfilaremia but no history of eye worm; EW = positive history of eye worm but no detectable microfilaria; EWMF = positive history of eye worm as well as detectable microfilaremia.

Hematological findings were analyzed following the same categorization and adjustments as described above (see Table D and E in S1 Appendix). Data for differential blood count were incomplete or missing for 133 (10.8%) participants. There was no statistical difference in hemoglobin values between LP and LN nor between the different loiasis infection states. However, LP had higher total WBC counts (median [inter quartile range]: 7.4 [5.9–8.9] vs. 6.7 [5.4–8.4] x10^3^ leucocytes/μL, p-adj.<0.001), as well as higher absolute eosinophils/μL (1.1 [0.5–1.8] vs. 0.6 [0.25–1.3] x10^3^, p-adj.<0.001) and relative eosinophil counts (15.0% [8.0–22.0] vs. 9.2% [4.1–17.0] of total WBCC, p-adj.<0.001) compared to LN. Differences in WBC counts and eosinophilia were also found when comparing the loiasis infection states individually to LN and among each other. Fig 2 displays the results graphically including adjusted p-values.

Comparing all individuals with microfilaremia (MF and EWMF) to patients with history of eye worm only (EW), the two former categories were characterized by higher absolute eosinophil (1.3 [0.8–1.85] and 1.4 [0.8–2.0] vs. 0.8 [0.34–1.6] 10^3^/μL; p-adj. = 0.076 and 0.003) and relative eosinophil counts (18.0% [11.0–23.0] and 17.0% [12.0–24.0] vs. 13.0% [6.0–21.0]; p-adj. = 0.016 and 0.002). To further investigate this association between microfilaremia and eosinophilia, comparisons were made between the microfilaremic groups. The HYMF group had higher absolute eosinophil counts than HMF and LMF (1.70 [0.60–2.70] vs.1.40 [0.90–1.90] and 1.30 [0.80–1.90] 10^3^/μL; p-adj. = 0.034 and p-adj. = 0.021), respectively (see Fig A in S1 Appendix). Of note, of the 56 individuals in the HMF and HYMF groups, none had an absolute eosinophil count within normal range (≤0.5x10^3^/μL). Next, a simple linear regression analysis of the microfilaremic sub-population of the study, with at least 2 microfilariae per mL revealed an increase in absolute eosinophil count with every 10-fold increase in parasitemia (p-adj. = 0.012, ß-estimate: 0.17 [0.04–0.31], see Fig B in S1 Appendix).

In summary, loiasis was associated with absolute and relative eosinophilia. Extent of eosinophilia was higher in microfilaremic individuals in comparison to only eye worm positives. The level of eosinophilia was associated with the quantity of *L*. *loa* microfilaremia.

## Discussion

**Fig 3:**
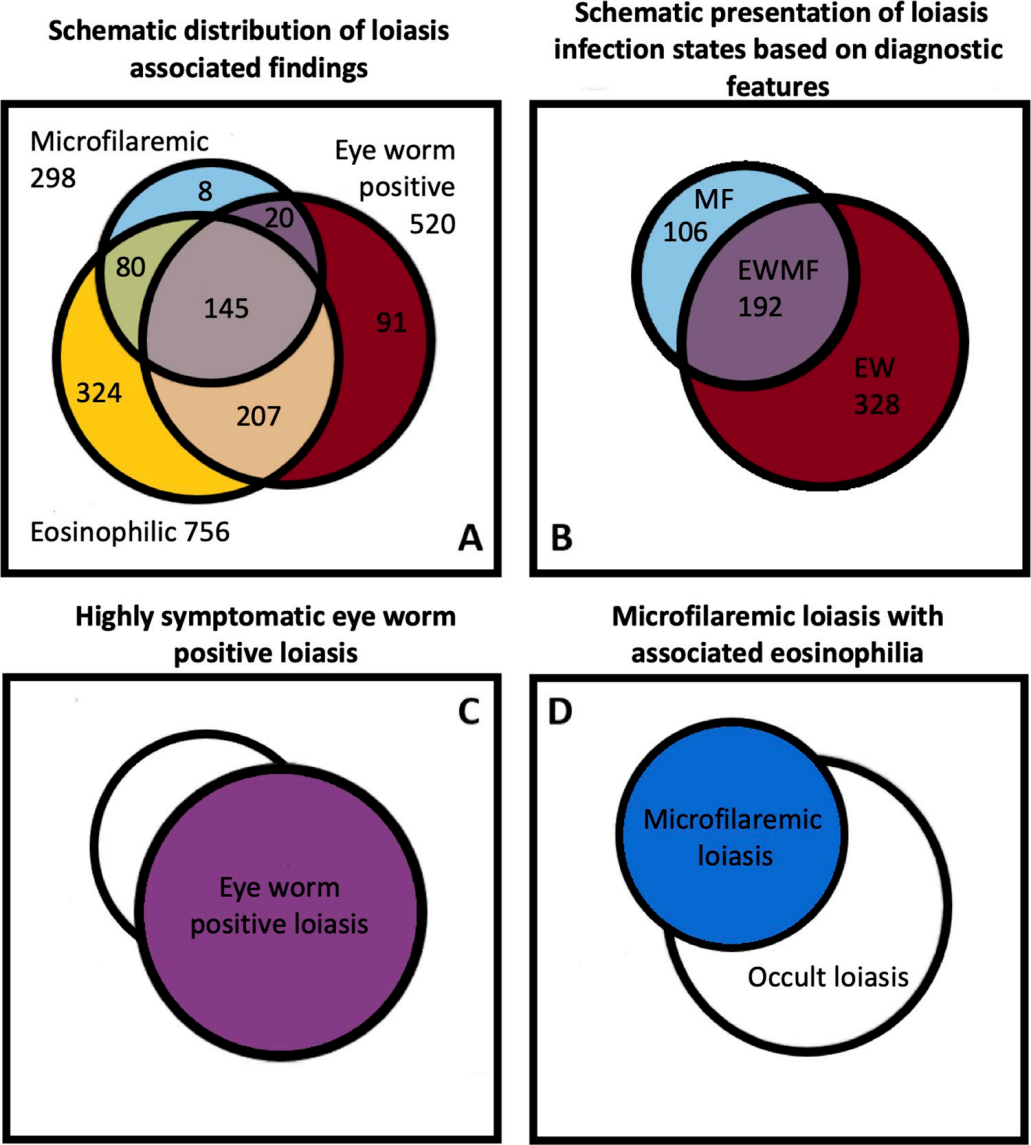
Graphic presentation of the hematological and clinical findings within the study population. 3A is a diagram depicting the distribution and overlap of the diagnostic findings of history of eye worm, microfilaremia and eosinophilia in the study population (Eosinophilia data missing for 133 (10.8%) participants, see supporting information Table D). 3B depicts the distribution of the three mutually exclusive infection states in the same diagram based on diagnostic criteria. These include microfilaremic (MF), positive history of eye worm (EW), and individuals positive for both (EWMF). 3C displays the clinical outcome of eye worm positive loiasis and 3D the hematological outcome of microfilaremic loiasis in this schematic display. Note that these outcomes are not mutually exclusive, but overlap in individuals who are positive for both diagnostic criteria.

Here we describe the clinical and hematological findings associated with *L*. *loa* infection in a large population of individuals living in a highly endemic region. We found that distinct infection states of loiasis were associated with specific clinical and hematological findings (see Fig 3A). The loiasis positive infection states can be classified into three mutually exclusive categories based on diagnostic criteria. These categories consist of individuals having a positive history of eye worm (EW), microfilaremic individuals (MF) and individuals having both (EWMF) (Fig 3B). The associated clinical and hematological outcomes do overlap in individuals who had both diagnostic criteria but seem to be associated with the specific infection states (see Fig 3C and 3D).

Up to now clinical and hematological findings associated with loiasis have been described predominantly in travelers and temporary residents of endemic regions. However, differences in the clinical and immunological outcomes between short term residents and individuals living in endemic regions, as in other parasitic infections, are evident [1,3,4,7–13,15]. While it is known that loiasis causes various manifestations, including signs and symptoms of a rather “allergic type” and varying degrees of immune-activation, these effects have been described to be more predominant in travelers and short term residents than in residents of endemic regions with chronic infections [10,11]. However, knowledge on disease outcomes in individuals living in endemic regions is scarce and improved understanding of the disease in this patient population is now increasingly warranted [8–10,19,22–26].

In the here presented comprehensive dataset, participants of both sexes were found to be similarly often infected with *L*. *loa*, however microfilaremic loiasis was found disproportionally more often in men and only eye worm positive loiasis in women. Within the adult population the occurrence and distribution of microfilaremia remained rather stable in the different age groups, a pattern that has repeatedly been described and seems to be consistent across various endemic areas [1,5,27].

Comparing the loiasis infection states between each other showed that history of eye worm migration was associated with the presence of a wide range of signs and symptoms, independently of presence of microfilaremia. At the same time, individuals reporting only history of eye worm had only moderate elevation of absolute eosinophil counts. Consequently, individuals having a high symptom load, often lack diagnostic features of infection in the blood which would facilitate definitive diagnosis, such as microfilaremia or extensive eosinophilia. This constellation may hamper diagnosis, and could cause inappropriate clinical management and treatment while these patients are indeed in need of appropriate clinical care to improve their symptoms. Additionally, these findings underline that the omission of patients reporting only history of eye worm does not only significantly underestimate the true disease prevalence, but also the extent of associated symptoms, and thus the perceived and calculated associated disease burden [1,4,14,18,24]. Importantly, we found that neurological symptoms, including paresthesia and transient paralysis of extremities, were strongly associated with loiasis, specifically with eye worm positive loiasis and not with microfilaremia itself. While these symptoms have been described in reports on returning travelers, data on their occurrence in individuals living in endemic regions are scarce [7,28,29]. However, in the here described population these neurologic findings were quite frequent in the loiasis positive individuals and should thus be further investigated in future clinical studies. In addition to the neurological symptoms, a range of unspecific symptoms, such as fatigue or headache, were quite common and should therefore be taken into account by clinicians as diagnostic hints for loiasis.

Contrarily, microfilaremic individuals reported signs and symptoms in a similar frequency as individuals with no sign of loiasis infection, indicating that microfilaremia was not in itself associated with the queried symptoms. A potential explanation for this unapparent disease presentation could be a more Th-2 driven immune response leading to a lack of “reactive” symptoms in these individuals. A small study comparing microfilaremic to amicrofilaremic individuals provided data supporting this hypothesis, but clearly, this theory still needs to be investigated in future studies [22].

Interestingly, in more historic descriptions of loiasis, when only microfilaremic individuals where diagnosed and eye worm positive loiasis was not taken into account, the infection was described to be rather asymptomatic—an observation that still shapes the perception of this disease today [7,18,19,24]. The here described finding, that microfilaremia per se is not associated with symptoms, could thus serve as an explanation for this discrepancy between current and historical disease descriptions.

Eosinophils are paramount for host defense against helminth infections and significant eosinophilia can be caused by various helminthic pathogens [30,31]. In loiasis, differences in the extent of eosinophilia and eosinophil activation have been noted between temporary residents and residents of endemic regions [8–11]. While *L*. *loa*-associated eosinophilia has been described in smaller cohorts, to our knowledge this is the first analysis of eosinophilia by distinct loiasis infection states in a large group of individuals from an endemic region [13]. Here, all loiasis positive infection states were associated with higher absolute eosinophil counts compared to individuals without signs of loiasis. Importantly, eosinophilia was higher in microfilaremic individuals than in amicrofilaremic loiasis. Furthermore, the extent of eosinophilia seemed to be associated with *L*. *loa* microfilarial density and, conclusively all 56 individuals with ≥8,000mf/ml, had eosinophil counts above the normal range (≤0.5x10^3^/μL). The difference of absolute eosinophil counts between individuals with and without signs of loiasis and between microfilaremic and amicrofilaremic loiasis patients was statistically significant and may also be of clinical relevance. Of note, microfilaremia with related blood eosinophilia was not associated with subjective symptoms.

It is established that chronic eosinophilia can cause organ damage due to chronic inflammation [32]. The here described finding of an association between extent of eosinophilia and microfilaria counts, might therefore pathophysiologically explain the previously described excess mortality in highly microfilaremic individuals [13,19]. However, this theory needs further investigation to be verified or refuted.

Further, one could hypothesize that combining the subjective well-being of hyper-microfilaremic individuals and their previously described risk for excess mortality, could lead to inappropriately low health care seeking behavior in those most at risk for severe disease outcomes.

An important limitation of our analysis is the potential confounding of eosinophilia by other parasitic infections prevalent in the study region [3,15,20]. But, despite this co-endemicity of various parasites, hematological changes were consistently found to be associated with different infection states of loiasis, supporting the hypothesis that eosinophilia is indeed primarily caused or at least significantly augmented by *L*. *loa* infection. In line with this data, is the fact that the occurrence and extent of eosinophilia has long been known to depend on infection states in other filarial diseases, such as lymphatic filariasis and onchocerciasis, further supporting the here presented findings [33–35].

To understand the relevance of eosinophilia in loiasis in more detail, more clinical and immunological data are needed, especially as it is known that the provoked pathology depends on the activation of these effector cells [36]. Different immune activation mechanisms may underlie the different disease manifestations. Further longitudinal studies investigating the association of specific immune responses, including eosinophilic activation markers with infection states, would therefore be of interest.

Importantly, the cross-sectional study design does not allow a direct inference of a cause-effect relationship between associated factors, nor the analysis of natural evolution of single versus repeated infections or assessments of more clinically serious outcomes such as organ lesions, organ damage or death [37]. Of note, due to the cross-sectional study design, laboratory findings collected at a single time point, including microfilaremia and eosinophilia, and retrospectively reported findings, including history of eye worm and occurrence of symptoms, were compared. This is a clear limitation of this study. Therefore, it is evident that the reported findings and raised hypotheses need to be addressed in prospective, clinical studies for further verification. However, due to the long duration of infections and the rare appearance of worms in the eye—a major diagnostic criterion—longitudinal studies of loiasis are extremely challenging.

In general, there are several pitfalls in loiasis diagnosis and associated difficulties have repeatedly been discussed [38]. Even in individuals where no microfilariae can be found, it is important to state that microfilaremia might still be present but just below the limit of detection of the used method. Here we used a combination of microscopy of thick blood smears and an additional concentration step assessing 1 mL of blood in order to raise the sensitivity of microfilariae detection. A qPCR based on previously published methods by Fink et al., 2011 was performed on all samples, but did not improve sensitivity and therefore results were not used for classification in this analysis (manuscript in preparation) [39]. However, very low microfilaremic individuals might have been missed and thus might have been wrongly classified as individuals without microfilaremia.

Serology has been shown to be cross-reactive and seroprevalence is very high in the study population as shown by a recent analysis (manuscript under preparation). Thus, currently available serological methods seem inappropriate to definitively distinguish active from past infection and counting all seropositive individuals as loiasis positive would likely overestimate the true disease prevalence [38,40,41]. In the absence of an adequate diagnostic tool, proving or refuting an active infection in amicrofilaremic individuals is difficult, as adult filaria might not always become evident as eye worm. In fact, it is not known which percentage of actively infected individuals never experience an eye worm migration. On the other hand, it is not known how long after a reported eye worm migration an individual remains actively infected. While it would be possible that the parasite died shortly after appearing as an eye worm, based on clinical reports it is estimated that adult *L*. *loa* filaria can live more than 15 years in the host [23]. Combining these factors, it becomes evident that history of eye worm is an imprecise diagnostic feature and does not allow to differentiate active from past infection. This may have led to misclassifications of individuals into incorrect infection states in the presented analysis. Nevertheless, the standardized questionnaire for history of eye worm (RAPLOA) remains one of the most important diagnostic tools for loiasis until better methods for detection of occult loiasis are developed. Therefore, while not allowing to exclude an infection, RAPLOA was used together with microscopy for microfilaremia, for diagnosis of loiasis in this study [2]. In conclusion, while there are obvious limitations of the data due to the cross-sectional study design, our findings are based on the best available diagnostic methods.

Further, the here provided findings were described in a large group of individuals who are likely to be re-exposed and chronically infected over several years. This underlines the significant clinical and hematological impact of loiasis, and refute once more the notion of loiasis being an inconspicuous, asymptomatic infection [1,7,12–14,19,23,37]. Contrarily, loiasis apparently shares important similarities to other filarial diseases, for which the respective infection states leading to various disease outcomes have long been acknowledged [34,35].

These data show that loiasis is a complex disease, manifesting with distinct clinical and hematological outcomes in individuals living in a highly endemic region. The consequences of these findings for the affected individuals or on populations as well as the pathophysiological consequences of correlating eosinophilia are largely unknown. Currently, loiasis remains highly neglected and individuals in endemic regions are often left untreated [18,19,24,25]. High-quality research on loiasis needs to be further fostered to provide adequate treatment options for the most affected populations and to drive the development of adequate elimination strategies.

## Supporting information

S1 Appendix Table A: Proportion of reported manifestations according to infection states. Table B: Intergroup comparisons of the proportion of reported disease manifestations in respective infection states. Table C: Intergroup comparisons of the proportion of reported disease manifestations in microfilaria density groups. Table D: Overview on hemoglobin levels and differential blood count according to different infection states of loiasis. Table E: Intergroup comparisons of hemoglobin levels and differential blood count according to different infection states of loiasis. Supporting information PCR Methods. Fig A: Boxplot displaying the eosinophilia distribution by microfilaremia group (* indicates a p-adj.<0.01). Fig B: Regression plot showing the association between microfilaremia and eosinophilia.
(DOCX)

S2 Appendix This file contains the data. (XLSX)
