# Supplementary material for: Distinct loiasis infection states and associated clinical and hematological manifestations in patients from Gabon
Source: PLoS Negl Trop Dis. 2022 Sep 19;16(9):e0010793. doi: 10.1371/journal.pntd.0010793 (PMC9521832; doi:10.1371/journal.pntd.0010793)
Supplement: S1 Appendix — Table A: Proportion of reported manifestations according to infection states. Table B: Intergroup comparisons of the proportion of reported disease manifestations in respective infection states. Table C: Intergroup comparisons of the proportion of reported disease manifestations in microfilaria density groups. Table D: Overview on hemoglobin levels and differential blood count according to different infection states of loiasis. Table E: Intergroup comparisons of hemoglobin levels and differential blood count according to different infection states of loiasis. Supporting information PCR Methods. Fig A: Boxplot displaying the eosinophilia distribution by microfilaremia group (* indicates a p-adj.<0.01). Fig B: Regression plot showing the association between microfilaremia and eosinophilia. (DOCX) [file pntd.0010793.s001.docx]

Table A: Proportion of reported manifestations according to infection states.

|  |  | overall, N (%) | LN*, n (%) | MF**, n (%) | EW***, n (%) | EWMF****, n (%) | p-value (Chi-2) |
| --- | --- | --- | --- | --- | --- | --- | --- |
| N |  | 1,232 (110) | 606 (49.2) | 106 (8.6) | 328 (26.6) | 192 (15.6) |  |
| Symptoms |  | Symptomfrequency within group (%) | | | | |  |
|  |  |  |  |  |  |  |  |
| Arthralgia | no | 769 (62.5) | 423 (69.9) | 66 (62.3) | 181 (55.2) | 99 (51.6) | <0.001 |
|  | yes | 462 (37.5) | 182 (30.1) | 40 (37.7) | 147 (44.8) | 93 (48.4) |  |
| Myalgia | no | 893 (72.7) | 472 (78.0) | 82 (77.4) | 216 (66.1) | 123 (64.4) | <0.001 |
|  | yes | 336 (27.3) | 133 (22.0) | 24 (22.6) | 111 (33.9) | 68 (35.6) |  |
| Pruritus | no | 546 (44.3) | 272 (44.9) | 58 (54.7) | 134 (40.9) | 82 (42.7) | 0.089 |
|  | yes | 686 (55.7) | 334 (55.1) | 48 (45.3) | 194 (59.1) | 110 (57.3) |  |
| Calabar swelling during the previous year | no | 784 (63.9) | 432 (71.5) | 76 (71.7) | 188 (57.7) | 88 (46.1) | <0.001 |
|  | yes | 443 (36.1) | 172 (28.5) | 30 (28.3) | 138 (42.3) | 103 (53.9) |  |
| Random swellings | no | 1,001 (81.9) | 517 (86.0) | 90 (85.7) | 244 (75.1) | 150 (78.5) | <0.001 |
|  | yes | 221 (18.1) | 84 (14.0) | 15 (14.3) | 81 (24.9) | 41 (21.5) |  |
| Urticaria | no | 1032 (83.8) | 519 (85.6) | 96 (90.6) | 255 (78.0) | 162 (84.4) | 0.004 |
|  | yes | 199 (16.2) | 87 (14.4) | 10 (9.4) | 72 (22.0) | 30 (15.6) |  |
| Paresthesia | no | 872 (73.5) | 458 (79.4) | 80 (77.7) | 215 (67.4) | 119 (63.6) | <0.001 |
|  | yes | 314 (26.5) | 119 (20.6) | 23 (22.3) | 104 (32.6) | 68 (36.4) |  |
| Paralysis | no | 1,036 (84.6) | 545 (90.4) | 95 (89.6) | 214 (74.6) | 155 (80.7) | <0.001 |
|  | yes | 188 (15.4) | 58 (9.6) | 11 (10.4) | 82 (25.4) | 37 (19.3) |  |
| Severe headache | no | 631 (51.5) | 343 (56.9) | 60 (57.1) | 134 (41.1) | 94 (49.0) | <0.001 |
|  | yes | 595 (48.5) | 260 (43.1) | 45 (42.9) | 192 (58.9) | 98 (51.0) |  |
| Fatigue | no | 829 (67.7) | 460 (76.0) | 73 (69.5) | 180 (55.7) | 116 (60.7) | <0.001 |
|  | yes | 395 (32.3) | 145 (24.0) | 32 (30.5) | 143 (44.3) | 75 (39.3) |  |
| * LN = No sign of loiasis infection, **MF = detectable microfilaremia but no history of eye worm, ***EW = positive history of eye worm but no detectable microfilaria, **** EWMF = positive history of eye worm as well as detectable microfilaremia, % = column percentages | | | | | | | |

Table B: Intergroup comparisons of the proportion of reported disease manifestations in respective infection states.

| inter group comparisons | | | | | | | | | |
| --- | --- | --- | --- | --- | --- | --- | --- | --- | --- |
|  | groups | adj. p value* | aPR* (95% CI) | groups | adj. p value* | aPR* (95% CI) | groups | adj. p value* | aPR* (95% CI) |
| Arthralgia | MF/LN | 1.000 | 1.00 (0.64-1.55) | EW/MF | 0.291 | 1.27 (0.81-2.00) | EW/EWMF | 0.688 | 0.93 (0.66-1.32) |
|  | EW/LN | 0.088 | 1.27 (0.97-1.67) | EWMF/MF | 0.196 | 1.37 (0.85-2.19) |  |  |  |
|  | EWMF/LN | 0.067 | 1.37 (0.98-1.90) |  |  |  |  |  |  |
| Myalgia | MF/LN | 0.273 | 0.76 (0.46-1.25) | EW/MF | 0.023 | 1.79 (1.08-2.97) | EW/EWMF | 0.923 | 1.02 (0.71-1.46) |
|  | EW/LN | 0.041 | 1.36 (1.01-1.82) | EWMF/MF | 0.035 | 1.76 (1.04-2.99) |  |  |  |
|  | EWMF/LN | 0.109 | 1.33 (0.94-1.89) |  |  |  |  |  |  |
| Pruritus | MF/LN | 0.279 | 0.93 (0.79-1.08) | EW/MF | 0.057 | 1.14 (0.98-1.33) | EW/EWMF | 0.815 | 0.99 (0.90-1.08) |
|  | EW/LN | 0.167 | 1.06 (0.98-1.14) | EWMF/MF | 0.051 | 1.15 (0.99-1.35) |  |  |  |
|  | EWMF/LN | 0.170 | 1.07 (0.98-1.17) |  |  |  |  |  |  |
| Calabar swelling | MF/LN | 0.639 | 0.90 (0.59-1.39) | EW/MF | 0.043 | 1.55 (1.00-2.40) | EW/EWMF | 0.01 | 0.68 (0.51-0.91) |
|  | EW/LN | 0.008 | 1.40 (1.10-1.79) | EWMF/MF | <0.001 | 2.27 (1.45-3.55) |  |  |  |
|  | EWMF/LN | <0.001 | 2.05 (1.56-2.69) |  |  |  |  |  |  |
| Random swellings | MF/LN | 0.901 | 0.97 (0.56-1.66) | EW/MF | 0.025 | 1.81 (1.05-3.10) | EW/EWMF | 0.283 | 1.21 (0.85-1.74) |
|  | EW/LN | <0.001 | 1.75 (1.31-2.34) | EWMF/MF | 0.164 | 1.49 (0.84-2.63) |  |  |  |
|  | EWMF/LN | 0.060 | 1.44 (1.00-2.08) |  |  |  |  |  |  |
| Urticaria | MF/LN | 0.161 | 0.63 (0.33-1.22) | EW/MF | 0.007 | 2.38 (1.23-4.59) | EW/EWMF | 0.062 | 1.48 (0.97-2.24) |
|  | EW/LN | 0.01 | 1.50 (1.11-2.03) | EWMF/MF | 0.176 | 1.61 (0.80-3.24) |  |  |  |
|  | EWMF/LN | 0.929 | 1.02 (0.67-1.55) |  |  |  |  |  |  |
| Paresthe-sia | MF/LN | 0.836 | 1.05 (0.68-1.60) | EW/MF | 0.079 | 1.44 (0.94-2.22) | EW/EWMF | 0.343 | 0.88 (0.67-1.15) |
|  | EW/LN | 0.001 | 1.51 (1.19-1.92) | EWMF/MF | 0.019 | 1.65 (1.07-2.56) |  |  |  |
|  | EWMF/LN | <0.001 | 1.73 (1.32-2.26) |  |  |  |  |  |  |
| Paralysis | MF/LN | 0.822 | 0.93 (0.47-1.81) | EW/MF | 0.001 | 2.84 (1.48-5.47) | EW/EWMF | 0.059 | 1.48 (0.98-2.23) |
|  | EW/LN | <0.001 | 2.63 (1.86-3.72) | EWMF/MF | 0.06 | 1.92 (0.97-3.83) |  |  |  |
|  | EWMF/LN | 0.011 | 1.78 (1.15-2.76) |  |  |  |  |  |  |
| Severe headache | MF/LN | 0.618 | 1.07 (0.83-1.38) | EW/MF | 0.078 | 1.24 (0.96-1.61) | EW/EWMF | 0.173 | 1.14 (0.94-1.38) |
|  | EW/LN | <0.001 | 1.33 (1.15-1.54) | EWMF/MF | 0.530 | 1.09 (0.83-1.44) |  |  |  |
|  | EWMF/LN | 0.131 | 1.17 (0.97-1.41) |  |  |  |  |  |  |
| Fatigue | MF/LN | 0.419 | 1.18 (0.79-1.77) | EW/MF | 0.033 | 1.53 (1.02-2.30) | EW/EWMF | 0.20 | 1.21 (0.90-1.63) |
|  | EW/LN | <0.001 | 1.81 (1.43-2.30) | EWMF/MF | 0.278 | 1.27 (0.82-1.94) |  |  |  |
|  | EWMF/LN | 0.01 | 1.49 (1.12-2.01) |  |  |  |  |  |  |
| LN = No sign of loiasis infection, MF = detectable microfilaremia but no history of eye worm, EW = positive history of eye worm but no detectable microfilaria, EWMF = positive history of eye worm as well as detectable microfilaremia, aPR = adjusted prevalence ratio, * adjusted to sex, age and *M. perstans* PCR positivity | | | | | | | | | |

Table C: Intergroup comparisons of the proportion of reported disease manifestations in microfilaria density groups.

| Symptoms | LMF°, n (%) | HMF°°, n (%) | HYMF°°°, n (%) |  |  |  |  |
| --- | --- | --- | --- | --- | --- | --- | --- |
|  | 242 (81.2) | 43 (14.4) | 13 (4.4) |  | inter group comparisons | | |
|  | Symptom frequency within group (%) | | | p-value (Chi-2) | groups | adj. p-value* | aPR* (95% CI) |
| Arthralgia | 132 (54.5) | 25 (58.1) | 8 (61.5) | 0.819 | HMF/LMH | 0.514 | 0.80 (0.40-1.58) |
|  | 110 (45.5) | 18 (41.9) | 5 (38.5) |  | HYMF/LMH | 0.481 | 0.65 (0.20-2.14) |
|  |  |  |  |  | HYMF/HMF | 0.765 | 0.81 (0.22-3.05) |
| Myalgia | 167 (69.3) | 29 (67.4) | 9 (69.2) | 0.971 | HMF/LMH | 0.914 | 1.03 (0.57-1.87) |
|  | 74 (30.7) | 14 (32.6) | 4 (30.8) |  | HYMF/LMH | 0.979 | 0.99 (0.35-2.76) |
|  |  |  |  |  | HYMF/HMF | 0.936 | 0.95 (0.31-2.97) |
| Pruritus | 114 (47.1) | 19 (44.2) | 7 (53.8) | 0.826 | HMF/LMH | 0.482 | 1.02 (0.96-1.08) |
|  | 128 (52.9) | 24 (55.8) | 6 (46.2) |  | HYMF/LMH | 0.771 | 0.98 (0.87-1.11) |
|  |  |  |  |  | HYMF/HMF | 0.524 | 0.97 (0.84-1.11) |
| Calabar swelling | 132 (54.8) | 24 (55.8) | 8 (61.5) | 0.889 | HMF/LMH | 0.344 | 0.76 (0.42-1.38) |
|  | 109 (45.2) | 19 (44.2) | 5 (38.5) |  | HYMF/LMH | 0.429 | 0.67 (0.24-1.89) |
|  |  |  |  |  | HYMF/HMF | 0.828 | 0.88 (0.29-2.73) |
| Random swellings | 192 (80.0) | 36 (83.7) | 12 (92.3) | 0.485 | HMF/LMH | 0.447 | 0.73 (0.32-1.66) |
|  | 48 (20.0) | 7 (16.3) | 1 (7.7) |  | HYMF/LMH | 0.261 | 0.33 (0.04-2.45) |
|  |  |  |  |  | HYMF/HMF | 0.451 | 0.45 (0.05-3.71) |
| Urticaria | 210 (86.8) | 36 (83.7) | 12 (92.3) | 0.713 | HMF/LMH | 0.709 | 1.14 (0.57-2.28) |
|  | 32 (13.2) | 7 (16.3) | 1 (7.7) |  | HYMF/LMH | 0.555 | 0.59 (0.09-3.71) |
|  |  |  |  |  | HYMF/HMF | 0.475 | 0.52 (0.08-3.48) |
| Paresthesia | 158 (67.2) | 32 (76.2) | 9 (69.2) | 0.514 | HMF/LMH | 0.219 | 0.73 (0.41-1.30) |
|  | 77 (32.8) | 10 (23.8) | 4 (30.8) |  | HYMF/LMH | 0.796 | 0.91 (0.42-1.96) |
|  |  |  |  |  | HYMF/HMF | 0.647 | 1.24 (0.50-3.05) |
| Paralysis | 204 (84.3) | 34 (79.1) | 12 (92.3) | 0.484 | HMF/LMH | 0.394 | 1.29 (0.73-2.29) |
|  | 38 (15.7) | 9 (20.9) | 1 (7.7) |  | HYMF/LMH | 0.449 | 0.52 (0.08-3.22) |
|  |  |  |  |  | HYMF/HMF | 0.294 | 0.40 (0.06-2.63) |
| Severe headache | 121 (50.2) | 24 (55.8) | 9 (69.2) | 0.349 | HMF/LMH | 0.322 | 0.85 (0.59-1.24) |
|  | 120 (49.8) | 19 (44.2) | 4 (30.8) |  | HYMF/LMH | 0.199 | 0.66 (0.29-1.49) |
|  |  |  |  |  | HYMF/HMF | 0.503 | 0.77 (0.34-1.76) |
| Fatigue | 157 (65.4) | 21 (48.8) | 11 (84.6) | 0.032 | HMF/LMH | 0.074 | 1.49 (0.95-2.33) |
|  | 83 (34.6) | 22 (51.2) | 2 (15.4) |  | HYMF/LMH | 0.156 | 0.40 (0.10-1.64) |
|  |  |  |  |  | HYMF/HMF | 0.038 | 0.27 (0.06-1.20) |
| °LMF= low microfilaremic(<8,000mf/ml), °° HMF = highly microfilaremic (>/= 8000 - <20,000mf/ml), °°° HYMF = hyper microfilaremic (>20,000mf/ml), % = column percentages, aPR = adjusted prevalence ratio, * adjusted to sex, age and *M. perstans* PCR positivity | | | | | | | |

Table D: Overview on hemoglobin levels and differential blood count according to different infection states of loiasis.

|  |  | overall, n (%) | LN*, n (%) | LP°, n (%) | MF**, n (%) | EW***, n (%) | EWMF****, n (%) |
| --- | --- | --- | --- | --- | --- | --- | --- |
| N (row%) |  | 1,232 (100) | 606 (49.2) | 626 (50.8) | 106 (8.6) | 328 (26.6) | 192 (15.6) |
|  | n (%) | available data n (% of group), Median (IQR) | | | | | |
| Haemoglobin, mg/dl | 1,232 | 12.6 (11.6-13.8) | 12.5 (11.4-13.7) | 12.7 (11.8-14.1) | 12.85 (11.9-14.10) | 12.6 (11.6-13.9) | 12.85 (11.8-14.1) |
|  |  |  |  |  |  |  |  |
|  |  |  | 555 (91.6) | 559 (89.3) | 91 (85.8) | 299 (91.2) | 169 (88.0) |
| WBCC abs/ul | 1,114 (90.4) | 7 (5.6-8.7) | 6.7 (5.4-8.4) | 7.4 (5.9-8.9) | 7.6 (6.3-8.9) | 7.0 (5.7-8.6) | 7.7 (6.4-9.5) |
|  |  |  |  |  |  |  |  |
|  |  |  | 548 (90.4) | 551 (88.0) | 88 (83.0) | 298 (90.9) | 165 (85.9) |
| Eosinophils, abs/ul | 1099 | 0.81 (0.31-1.60) | 0.6 (0.25-1.3) | 1.1 (0.5-1.8) | 1.3 (0.8-1.85) | 0.8 (0.34-1.6) | 1.4 (0.8-2.0) |
|  |  |  |  |  |  |  |  |
|  |  |  | 549 (90.6) | 551 (88.0) | 88 (83.0) | 298 (90.9) | 165 (85.9) |
| Eosinophils, % | 1,100 (89.3) | 12.0 (6.0-20.0) | 9.2 (4.1-17.0) | 15.0 (8.0-22.0) | 18.0 (11.0-23.0) | 13.0 (6.0-21.0) | 17.0 (12.0-24.0) |
|  |  |  |  |  |  |  |  |
|  |  |  | 549 (90.6) | 551 (88.0) | 88 (83.0) | 298 (90.9) | 165 (85.9) |
| Neutrophils, abs/ ul | 1,100 (89.3) | 2.50 (1.96-3.10) | 2.4 (1.9-3.1) | 2.57 (2.0-3.1) | 2.52 (2.0-3.0) | 2.5 (1.93-3.2) | 2.6 (2.1-3.1) |
|  |  |  |  |  |  |  |  |
|  |  |  | 549 (90.6) | 551 (88.0) | 88 (83.0) | 298 (90.9) | 165 (85.9) |
| Neutrophils, % | 1,100 (89.3) | 36.0 (30.0-42-0) | 36.9 (30.0-43.0) | 35.55 (30.0-41.0) | 35.0 (27.6-40.0) | 36.0 (30.8-42.0) | 34.0 (30.0-40.0) |
|  |  |  |  |  |  |  |  |
|  |  |  | 555 (91.6) | 559 (89.3) | 91 (85.8) | 299 (91.2) | 169 (88.0) |
| Basophils, abs/ul | 1,114 (90.4) | 0.0 (0.0-0.03) | 0.0 (0.0-0.05) | 0.0 (0.0-0.0) | 0.0 (0.0-0.03) | 0.0 (0.0-0.02) | 0.0 (0.0-0.0) |
|  |  |  |  |  |  |  |  |
|  |  |  | 555 (91.6) | 559 (89.3) | 91 (85.8) | 299 (91.2) | 169 (88.0) |
| Basophils, % | 1,114 (90.4) | 0.0 (0.0-0.6) | 0.0 (0.0-0.08) | 0.0 (0.0-0.0) | 0.0 (0.0-0.06) | 0.0 (0.0-0.03) | 0.0 (0.0-0.0) |
| * LN = No sign of loiasis infection, °LP= all loiasis positives (MF+EW+EWMF), **MF = detectable microfilaremia but no history of eye worm, ***EW = positive history of eye worm but no detectable microfilaria, **** EWMF = positive history of eye worm as well as detectable microfilaremia, % = column percentages | | | | | | | |

Table E: Intergroup comparisons of hemoglobin levels and differential blood count according to different infection states of loiasis.

|  | LP compared to LN | inter group comparisons | | | | | |
| --- | --- | --- | --- | --- | --- | --- | --- |
|  | adj.p value* | groups | adj. p value* | groups | adj. p value* | groups | adj. p value* |
| Haemoglobin, mg/dl | 0.668 | MF/LN | 0.963 | EW/MF | 0.538 | EW/EWMF | 0.147 |
|  |  | EW/LN | 0.289 | EWMF/MF | 0.603 |  |  |
|  |  | EWMF/LN | 0.505 |  |  |  |  |
| WBCC abs/ul | <0.001 | MF/LN | 0.003 | EW/MF | 0.183 | EW/EWMF | 0.008 |
|  |  | EW/LN | 0.013 | EWMF/MF | 0.456 |  |  |
|  |  | EWMF/LN | <0.001 |  |  |  |  |
| Eosinophils, abs/ul | <0.001 | MF/LN | <0.001 | EW/MF | 0.076 | EW/EWMF | 0.003 |
|  |  | EW/LN | 0.013 | EWMF/MF | 0.579 |  |  |
|  |  | EWMF/LN | <0.001 |  |  |  |  |
| Eosinophils, % | <0.001 | MF/LN | <0.001 | EW/MF | 0.016 | EW/EWMF | 0.002 |
|  |  | EW/LN | 0.015 | EWMF/MF | 0.994 |  |  |
|  |  | EWMF/LN | <0.001 |  |  |  |  |
| Neutrophils, abs/ul | 0.011 | MF/LN | 0.339 | EW/MF | 0.745 | EW/EWMF | 0.553 |
|  |  | EW/LN | 0.038 | EWMF/MF | 0.458 |  |  |
|  |  | EWMF/LN | 0.023 |  |  |  |  |
| Neutrophils, % | 0.462 | MF/LN | 0.168 | EW/MF | 0.145 | EW/EWMF | 0.181 |
|  |  | EW/LN | 0.827 | EWMF/MF | 0.713 |  |  |
|  |  | EWMF/LN | 0.218 |  |  |  |  |
| Basophils, abs/ul | 0.749 | MF/LN | 0.949 | EW/MF | 0.859 | EW/EWMF | 0.09 |
|  |  | EW/LN | 0.693 | EWMF/MF | 0.272 |  |  |
|  |  | EWMF/LN | 0.141 |  |  |  |  |
| Basophils, % | 0.648 | MF/LN | 0.499 | EW/MF | 0.274 | EW/EWMF | 0.021 |
|  |  | EW/LN | 0.464 | EWMF/MF | 0.487 |  |  |
|  |  | EWMF/LN | 0.065 |  |  |  |  |
| LN = No sign of loiasis infection, LP= all loiasis positives (MF+EW+EWMF), MF = detectable microfilaremia but no history of eye worm, EW = positive history of eye worm but no detectable microfilaria, EWMF = positive history of eye worm as well as detectable microfilaremia, * adjusted to sex, age and *M. perstans* PCR positivity | | | | | | | |

Supporting information PCR Methods:

To reliably detect both *Mansonella perstans* and the genotype *Mansonella sp.Deux* a FRET assay followed by a melting curve analysis was designed, targeting an ITS region present in both Mansonella genotypes. The assay was designed with LightCycler Probe Design Software 2.0 by Roche with the Design Type Mutation HypProbe. PCR and melting curve analysis were done with 2ul DNA and 8ul mastermix using the following primers and settings:

| Primer | Mans-FRET-ITS-F 5'-CCTAAACCGTCGATAATGATGA-3' |
| --- | --- |
|  | Mans-FRET-ITS-R 5'-CACCGCTAAGAGTTAAAAATTTC-3' |
|  | Mans-FRET-ITS-S Cy5'-AATACACACATACATATACTAATTGTAATTATTGA-3' Phosphat |
|  | Mans-FRET-ITS-A 5'-AATAAGCATTTATGCTAAATATGCTACCAACAAAT-3' 6FAM |
| Mastermix | MgCl2, Solution S, Puffer BD, dNTPs, a HotStart Taq Polymerase (Solis) |
| PCR settings | 95°C 15min; (95°C 20sec, 60°C 45sec, 72°C 20sec) x 45cycles; 72°C 5min |
| Melting curve analysis | Temperature range: 35°C to 75°C |

**Fig. A : Boxplot displaying the eosinophilia distribution by microfilaremia group (* indicates a p-adj.<0.01).
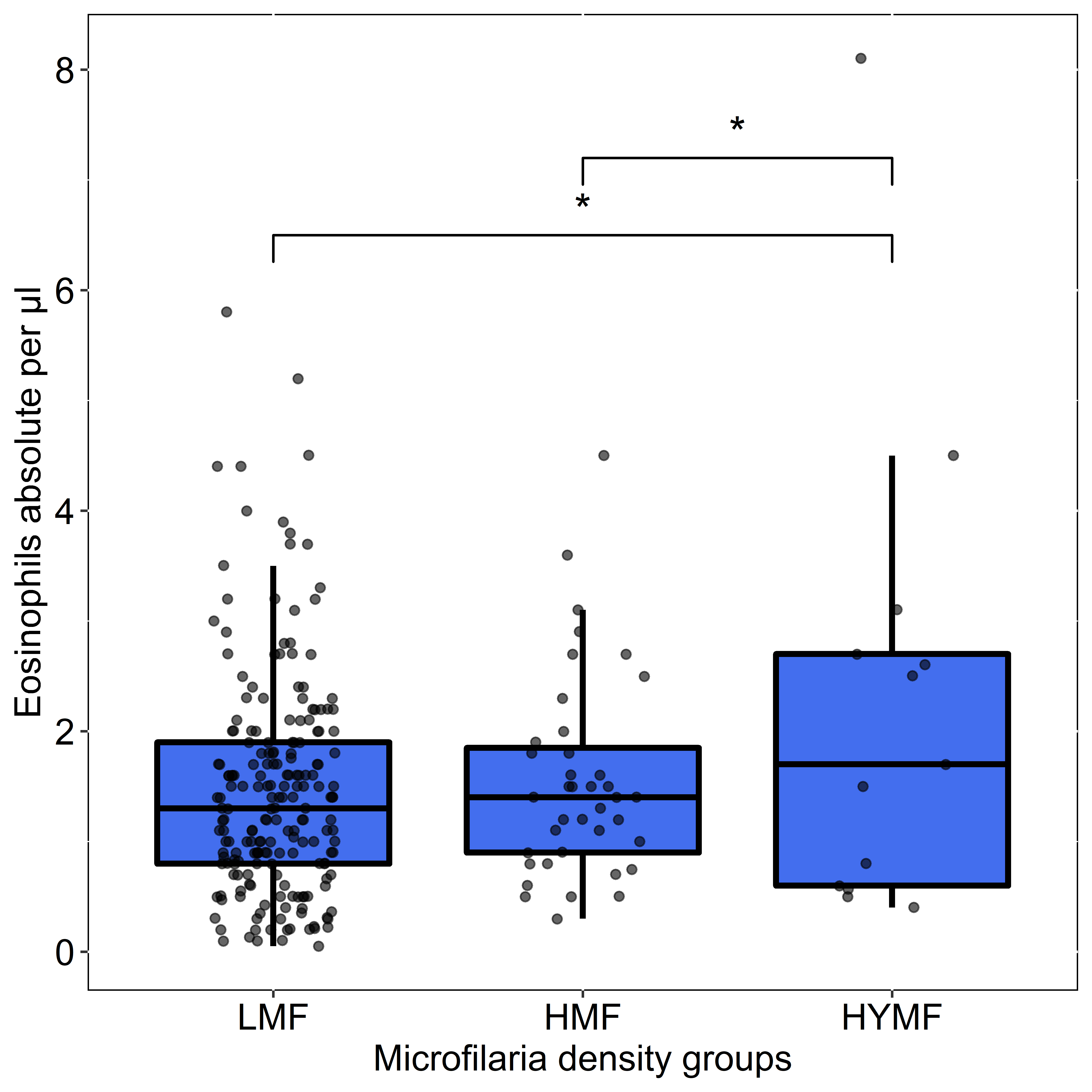
**

**Fig. B: Regression plot showing the association between microfilaremia and eosinophilia.**

**
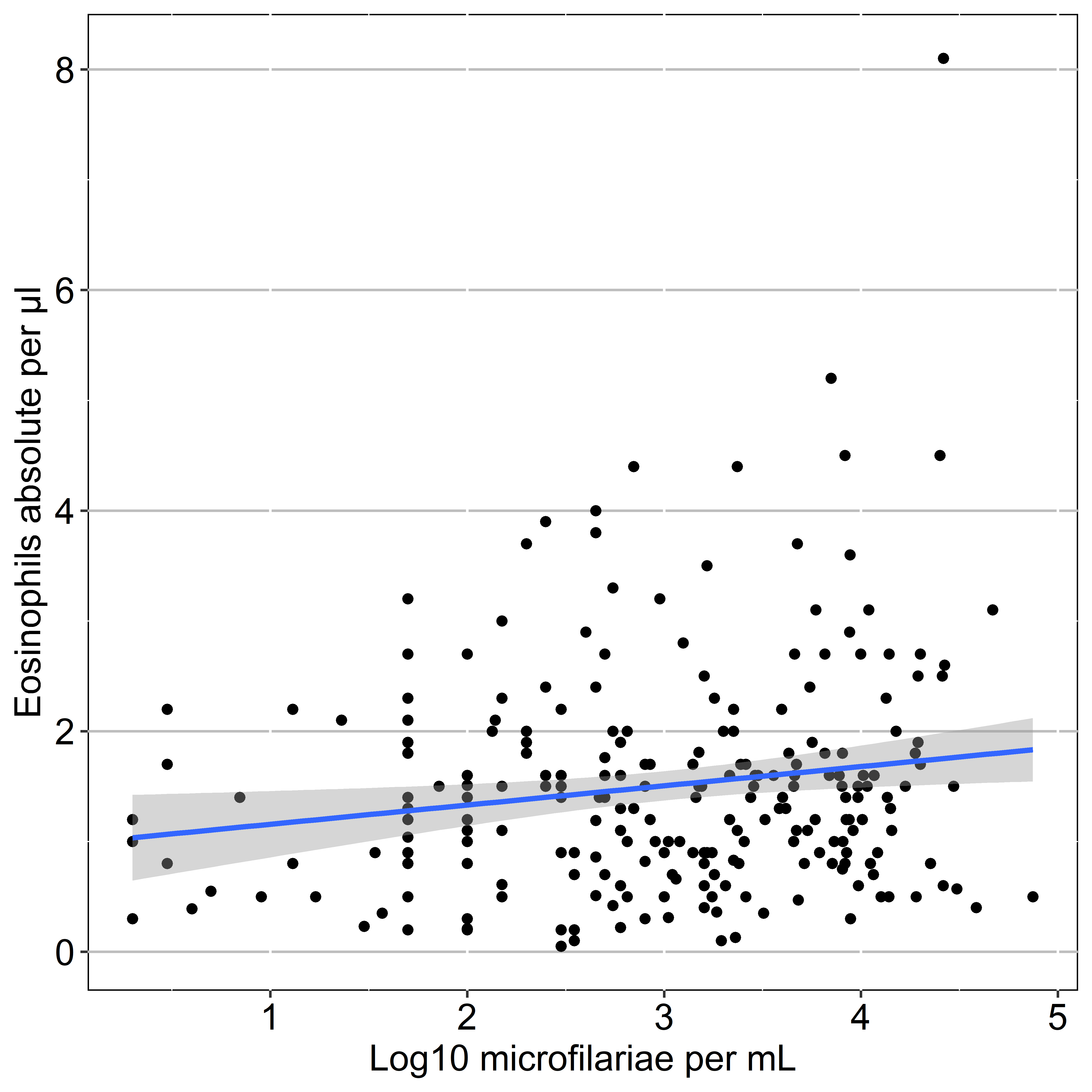
**
